# Prospective Assessment of Systemic MicroRNAs as Markers of Response to Neoadjuvant Chemotherapy in Breast Cancer

**DOI:** 10.3390/cancers12071820

**Published:** 2020-07-07

**Authors:** Andrew McGuire, Maire-Caitlin Casey, Ronan M. Waldron, Helen Heneghan, Olga Kalinina, Emma Holian, Ailbhe McDermott, Aoife J. Lowery, John Newell, Róisín M. Dwyer, Nicola Miller, Maccon Keane, James A.L. Brown, Michael J. Kerin

**Affiliations:** 1 Discipline of Surgery, School of Medicine, Lambe Institute for Translational Research, National University of Ireland Galway, Galway H91 YR71, Ireland; andrewmcguire@rcsi.ie (A.M.); mckc86@gmail.com (M.-C.C.); ronanwaldy@gmail.com (R.M.W.); helen.heneghan@hse.ie (H.H.); Ailbhe.McDermott@hse.ie (A.M.); aoife.lowery@nuigalway.ie (A.J.L.); roisin.dwyer@nuigalway.ie (R.M.D.); nicola.miller@nuigalway.ie (N.M.); 2 School of Mathematics, Statistics and Applied Mathematics, National University of Ireland Galway, Galway H91 TK33, Ireland; O.KALININA1@nuigalway.ie (O.K.); emma.holian@nuigalway.ie (E.H.); john.newell@nuigalway.ie (J.N.); 3 Department of Medical Oncology, Galway University Hospital, Galway H71 YR71, Ireland; maccon.keane@nuigalway.ie; 4 Department of Biological Sciences, University of Limerick, Limerick V94 T9PX, Ireland; 5 Centre for Chromosome Biology, School of Natural Science, National University of Ireland Galway, Galway H91 W2TY, Ireland; 6 Cancer Trials Ireland, Innovation House, Old Finglas Road, Dublin D11 KXN4, Ireland

**Keywords:** breast, microRNA, neoadjuvant, chemotherapy, prognostic, biomarker

## Abstract

Neoadjuvant chemotherapy (NACT) is used in locally advanced breast cancer to reduce tumour burden prior to surgical resection. However, only a subset of NACT treated patients will respond to treatment or achieve a pathologic complete response (pCR). This multicenter, prospective study (CTRIAL-IE (ICORG) 10-11 study) evaluated circulating microRNA as novel non-invasive prognostic biomarkers of NACT response in breast cancer. Selected circulating microRNAs (Let-7a, miR-21, miR-145, miR-155, miR-195) were quantified from patients undergoing standard of care NACT treatment (*n* = 114) from whole blood at collected at diagnosis, and the association with NACT response and clinicopathological features evaluated. NACT responders had significantly lower levels of miR-21 (*p* = 0.036) and miR-195 (*p* = 0.017), compared to non-responders. Evaluating all breast cancer cases miR-21 was found to be an independent predictor of response (OR 0.538, 95% CI 0.308–0.943, *p* < 0.05). Luminal cancer NACT responders were found to have significantly decreased levels of miR-145 (*p* = 0.033) and miR-21 (*p* = 0.048), compared to non-responders. This study demonstrates the prognostic ability of miR-21, miR-195 and miR-145 as circulating biomarkers stratifying breast cancer patients by NACT response, identifying patients that will derive the maximum benefit from chemotherapy.

## 1. Introduction

Adjuvant chemotherapy has been used in the management of breast cancer for several decades, and the current therapeutic protocols are dependent on the molecular subtype, disease stage and assessments of other clinicopathological factors influencing the potential for response (such as age and health). Neoadjuvant chemotherapy (NACT) is used in locally advanced breast cancer to reduce the tumour burden prior to surgery, increasing the number of patients suitable for breast conserving surgery. It also provides a unique opportunity for an in vivo assessment of how the tumour responds to chemotherapy. Pathological complete response (pCR) is the complete eradication of the tumour following the NACT treatment regimen. While studies have shown that pCR is associated with improved survival, this varies according to clinicopathological features and molecular subtype [1,2]. It has recently been reported that only 19.2% of patients will achieve a pCR [3]. The majority of patients exhibit only a partial or poor response to NACT, and currently there is no reliable, clinically validated biomarker to help clinicians stratify patients that will respond.

The introduction of molecular profiling resulted in the subdivision of breast cancer into at least 4 broad biologic subtypes, each with prognostic and therapeutic significance [4,5]. This insight into the fundamental molecular heterogeneity of breast cancer provides an explanation for the considerable variation observed in response to NACT. While limited molecular subtyping has been adopted into clinical practice to inform decision making in relation to therapeutic strategy, the comprehensive characterization of molecular subtypes requires whole genome profiling, and is not routinely performed in the clinical setting. The expression of the oestrogen receptor (OR), progesterone receptor (PR) and Her2/*neu* receptors (HER2+), which are routinely measured by immunohistochemistry (IHC), and are frequently used as practical, surrogate markers of the breast cancer biologic subtype [6].

Response to NACT has been shown to vary by breast cancer subtype, tumour grade and stage, with the highest complete response rates in the HER2+ (non-luminal) subtype [1,2,3]. Subtype specific therapy, such as trastuzumab, significantly increases the pCR rates in HER2 receptor positive breast cancers [7,8]. Response to NACT has a major impact on the risk of recurrence and survival, with patients that have a complete response having an over 90% five-year overall survival [1]. Although the role of chemotherapy in breast cancer is accepted, the majority of patients who get this treatment derive only a partial or no benefit, with non-responders having very poor outcomes [1,9]. Recently, non-invasive biomarkers found in blood (“liquid biopsies”) have been proposed as a possible way to not only distinguish breast cancer subtypes, but also to predict response to therapy. MiRNAs are short non-coding RNA, with a functional role in post-transcriptional regulation of gene expression [10,11]. Dysregulated miRNA expression has been shown in multiple cancers, and miRNA quantification for disease characterization is under investigation in clinical settings for many diseases [12,13,14]. Many miRNA, including targets investigated here, have previously been shown to be dysregulated (compared to healthy controls, either circulating and/or tumours), in breast cancer patients [15,16,17,18,19,20,21]. There is also evidence that miRNA expression/level profiles can accurately classify the breast cancer subtype, and can predict subtype specific survival [12,15,22,23,24]. Correspondingly, specific patterns of miRNA expression have been used to identify hormone receptor and HER2 receptor status [22]. Furthermore, it has been demonstrated that miRNA expression can effect response or resistance to systemic chemotherapy in breast cancer [25]. Recently, the profiling of circulating miRNA (ct miRNA found in plasma) to stratify NACT responders (from non-responders) in Her2+ patients (NeoALTTO study) has yielded four circulating miRNA signatures associated with pathologic complete response (pCR) [26].

The aim of our prospective translational study was to determine if any of a predefined panel of circulating miRNAs (Let-7a, miR-21, miR-145, miR-155, miR-195), extracted from whole blood collected at diagnosis, could predict NACT responders from non-responders. The secondary endpoint tested was to determine if selected circulating miRNA found in blood at breast cancer diagnosis could predict NACT response, in any of the four clinically relevant breast cancer subtypes.

## 2. Materials and Methods

### 2.1. Study Cohort and Disease Classification

Following ethical approval and informed patient consent, a multicentre, prospective translational study was established (CTRIAL-IE (ICORG) 10-11 study). Consecutive non-metastatic breast cancer patients undergoing the standard of care NACT for breast cancer were included. Clinicopathological details were obtained and recorded in a prospective database, with all patient details blinded from both investigators and research staff. All patients were aged 18 years or over, and gave written informed consent. Patients with distant metastatic disease at the time of the presentation were excluded. Response to NACT was based on the Miller–Payne classification, with patients who had a complete response or >90% reduction in primary tumour size (Grade 4 and 5) categorized as “responders”, while patients with <90% reduction in primary tumour size (Grades 1–3) were categorized as “non-responders”.

### 2.2. Breast Cancer Subtypes

Breast cancer subtypes were defined using OR, PR and HER2 receptor status. Luminal cancer is defined as (OR and/or PR+ve, HER2−ve), luminal B HER2 is defined as (OR and/or PR+ve, HER2+ve), HER2+(non-luminal) as (OR and PR−ve, HER2+ve), and triple negative as (OR and PR−ve, HER2−ve), according to the standard clinical pathological guidelines [4]. As Ki67 was not routinely reported, the luminal subtype could not be separated into true luminal A and luminal B. As per American Society of Clinical Oncology (ASCO) guidelines (ALLRED score >2, or more than 1% stain positive), the OR and PR receptor status were determined independently by clinical pathologists, as per standard clinical guidelines. The HER2 receptor status was identified by Herceptest^TM^ (DAKO Agilent pathology solutions, Santa Clara, CA, USA), with a score of 3+ considered to be positive. Any 2+ inconclusive results were confirmed using FISH (fluorescent in situ hybridization) testing, as per ASCO guidelines, with a HER2/CEP17 > 2.0 considered amplified.

### 2.3. Blood Collection and Analysis Cohort Details

Samples were collected from May 2011 to April 2014, from 8 centres across Ireland. Whole blood was collected at a single timepoint: at breast cancer diagnosis (in Ethylenediaminetetraacetic acid (EDTA) tubes), prior to standard of care NACT and surgery. From the total recruited cohort (*n* = 124), 9 samples were removed from miRNA analysis (no reliable detection of target miRNA), and 1 patient with grade 1 cancer was likewise removed from analysis (miRNA levels and cancer-related clinicopathological analysis). A study enrolment flow diagram illustrates the cohort recruitment and sample analysis workflow (Figure S1).

### 2.4. miRNA Panel

The levels of a panel of five miRNAs were selected for evaluation, on the basis of their reported relevance to breast cancer (Table 1) [15,16,17,18,19,20,22]. Two additional miRNAs (miR-16 and miR-425) were utilized as validated endogenous controls, having previously been demonstrated to be stably expressed in both breast cancer tissue, and have stable levels in the blood of breast cancer patients [18,19,27].

### 2.5. RNA Isolation

Total RNA was extracted from whole blood (1 mL) using Trizol (as per the manufacturer’s instructions). RNA concentrations were determined using spectrophotometry (NanoDrop ND-1000 Technologies Inc., Wilmington, DE, USA), as previously described [18].

### 2.6. RQ-PCR

TaqMan assays were used, as per the manufacturer’s instructions, for the relative quantification PCR (RQ-PCR) of the indicated target miRNA (miRNA: Taqman assay ID- miR-195: 000494; miR-155: 002623; miR-145: 002278; miR-21: 000397; Let-7a: 000377; miR-10b: 002218) and the endogenous control (miR-16: 000391; miR-425: 001104), as previously described (TaqMan Fast Universal Master Mix (2X), No AmpErase UNG: Applied biosystems, Foster City, CA, USA, cat:4367846) [18]. Assays were performed using an AB7900HT (Applied Biosystems), using standard conditions as per the manufacturer’s instructions. Moreover, miRNA expression levels were normalized using endogenous controls. All reactions were performed in triplicate (with each individual assay performed using technical triplicates). Raw fluorescence data from RQ-PCR were exported into the software package QBasePlus, and relative quantification was determined.

### 2.7. Analysis of miRNA Expression/Levels

Reliable detection of the five target miRNAs was performed on 114 at-diagnosis samples (*n* = 114). Moreover, miRNA expression levels were calculated using QbasePlus software (geNorm method), with results normalized to the two control miRNAs. For all miRNA (controls and targets) the threshold standard deviation for intra- and inter-assay replicates was 0.3. PCR amplification efficiencies were calculated for each candidate reference miRNA using the formula E = (10^−1/slope^ − 1) × 100, using the slope of the plot of quantification cycle (Cq) versus the log input of cDNA (10-fold dilution series). Notably, miRNA assays, and initial analysis, were performed blinded (to patient details).

### 2.8. Statistical Analysis

Data were analysed using R statistical software version 3.2.3. Non-parametric statistics were used, due to evidence of non-normally distributed data and non-ignorable outliers. The Kruskal–Wallis test was used to compare medians among multiple groups and the two-sample Wilcoxon rank sum test was used for all two-sample comparisons. A univariate logistic regression analysis was also performed. Results with a *p* value < 0.05 were considered statistically significant.

### 2.9. Ethical Approval

This study was conducted with ethical approval from Galway University Hospital and National University of Ireland Galway (approvals: C.A.151, 02/2008; and C.A.1012, 01/2014), and from the research ethics committees of the participating centres. All subjects gave their informed consent for inclusion before participation in the study.

### 2.10. Data Availability Statement

The data that support the findings of this study are available on request from the corresponding author. The data are not publicly available due to privacy or ethical restrictions.

## 3. Results

### 3.1. Patient Demographics

The clinicopathological details of the patient cohort are shown (Table 2), and the median age of patients was 55 years old (range 25–76). The luminal subtype was the most common subtype (*n* = 61, 49.2%), followed by triple negative (*n* = 25, 20.2%), luminal B HER2 (*n* = 22, 17.7%), with HER2+ (non-luminal) being the least common (*n* = 16, 12.9%). Following standard of care NACT, 45.2% (*n* = 56) of patients were found to be responders, with a complete pathological response seen in 25.8% (*n* = 32) of patients. As expected, the highest complete response rates (within each subtype) were seen in the HER2+ (non-luminal) breast cancer subtype (68.8%, *n* = 11), followed by triple negative (64%, *n* = 16), luminal B HER2 (59.1%, *n* = 13) and luminal (26.2%, *n* = 16), respectively.

### 3.2. Relationship of Circulating miRNA Levels to Clinicopathological Parameters

The association between the five target miRNAs and clinicopathological parameters, including grade, lymph node status at diagnosis, hormone receptor status, and HER2 receptor status is shown (Table 3). MiR-195 levels were significantly higher in grade 2 compared to grade 3 breast cancers (*p* = 0.016). Increased miR-195 levels were significantly associated with OR positive breast cancers (*p* = 0.014), while no significant variation in miR-195 levels was seen in relation to PR or HER2 receptor status. No significant variation in levels was seen in any of the variables for Let-7a, miR-21, miR-145 or miR-155 (Table 3).

**Figure 1:**
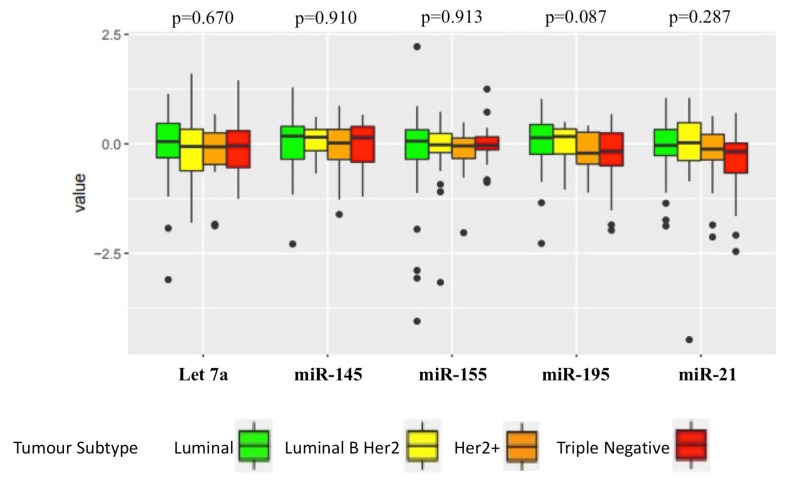
Levels of indicated miRNAs in the specified breast cancer subtypes. No significant association between the target miRNA level and any breast cancer subtype was found. Luminal, *n* = 57; Luminal B Her2, *n* = 20; Her2+, *n* = 14; Triple negative, *n* = 23.

Levels of the target miRNAs in each breast cancer subtype were then investigated (Figure 1). No significant differences in miRNA levels were seen between the breast cancer subtypes for any of the target miRNA (Let-7a, *p* = 0.670; miR-145, *p* = 0.910; miR-155, *p* = 0.913; miR-195, *p* = 0.087; miR-21, *p* = 0.287).

### 3.3. Relationship of Circulating miRNA in Responders versus Non-Responders

**Figure 2:**
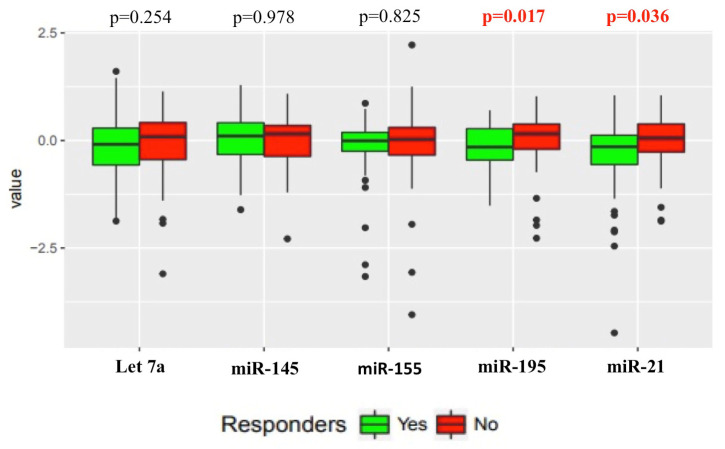
Relationship between the target miRNAs levels and tumour bed response to NACT in responders compared to non-responders. Responders *n* = 51, Non-responders *n* = 58. *p* < 0.05 considered significant.

The relationship between the target miRNAs’ levels and the tumour bed response to NACT in responders compared to non-responders was assessed. A significantly lower (*p* = 0.036) median level of miR-21 was seen in the responders (*n* = 51) compared to non-responders (*n* = 58). For miR-195, a significant difference (*p* = 0.017) in median level can also be seen between responders (*n* = 50) and non-responders (*n* = 59). No significant difference between responders and non-responders was seen for Let 7a (*p* = 0.254), miR-145 (*p* = 0.978) or miR-155 (*p* = 0.825) levels (Figure 2).

**Figure 3:**
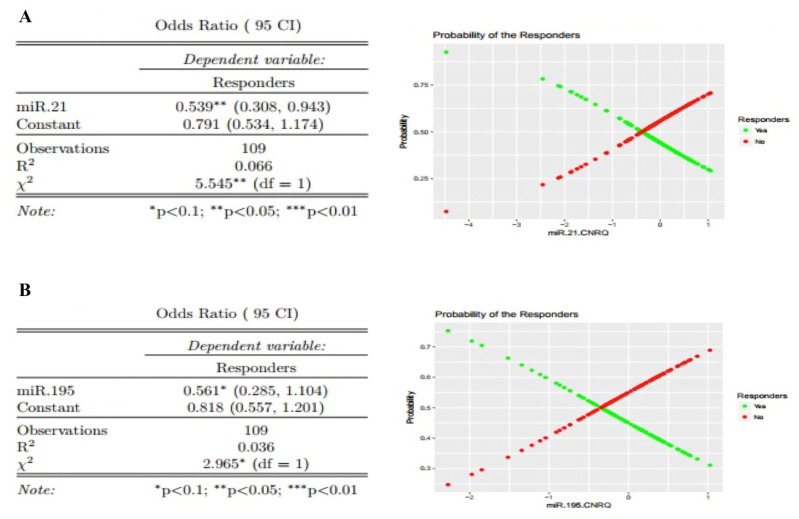
Univariate analysis of miRNA-21 and miRNA-195 level as an independent predictor of response. (**A**) For miRNA-21 every unit increase miRNA-21, the odds ratio of being a non-responder relative to a responder is 1.86 (1/0.538) times higher. (**B**) miRNA-195—with every unit increase in miRNA-195, the odds ratio of being a non-responder relative to a responder is 1.78 (1/0.561) times higher. Observations = *n* (indicated for each).

Using univariate logistic regression analysis, miR-21 was found to be an independent predictor of responders (OR 0.539, 95% CI 0.308–0.943, *p* < 0.05) (Figure 3A). For every unit increase in miR-21 levels, the odds ratio of being a non-responder versus a responder is 1.86 times higher. Using a univariate logistic regression analysis, for every unit increase in miR-195 levels, the odds ratio of being a non-responder relative to a responder is 1.78 times higher, however this was not found to be a significant effect (at significance level 0.05; OR 0.561, 95% CI 0.285–1.104, *p* < 0.1) (Figure 3B). Let 7a, miRNA-145 and miRNA-155 were not found to be predictors of responders (Figure S2). Investigating the sensitivity and specificity of the individual miRNA, only miR-195 displayed moderate diagnostic accuracy (Table S1).

### 3.4. Relationship of Individual Target miRNA Response to NACT in Different Breast Cancer Subtypes

**Figure 4:**
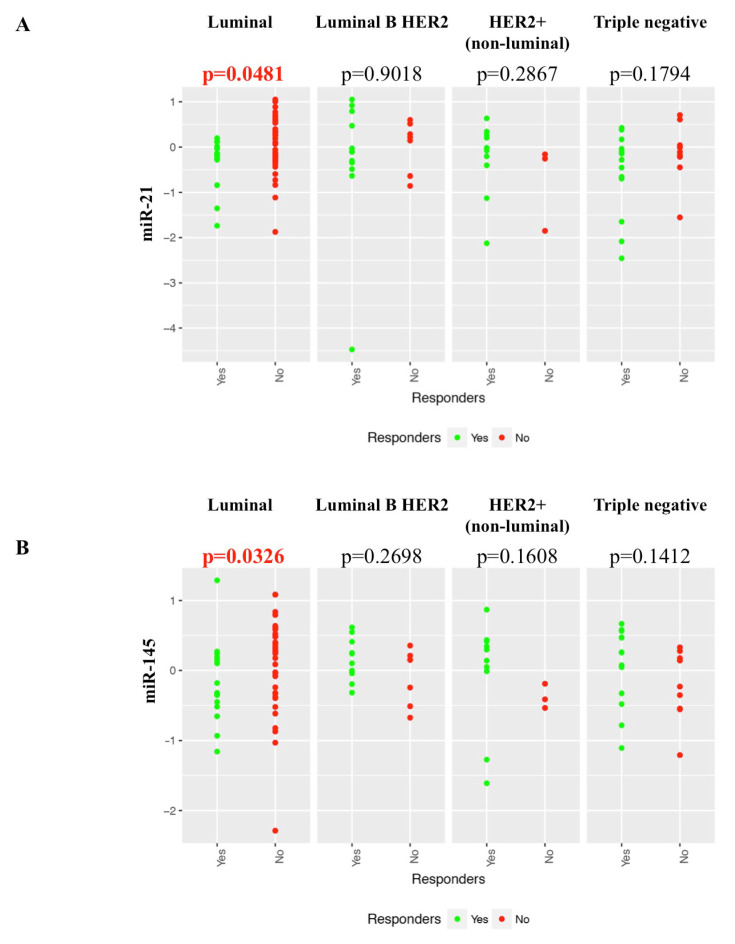
Variation in levels of each target miRNA by response to NACT was assessed in the four breast cancer subtypes. (**A**) miRNA-21 patients with low levels had a higher response rate in Luminal cancers. (**B**) miRNA-145 patients with low levels had a higher response rate in Luminal cancers. Luminal (responders *n* = 15, non-responders *n* = 41), Luminal B Her2 (responders *n* = 12, non-responders *n* = 7), Her2+ (non-luminal) (responders *n* = 10, non-responders *n* = 3), Triple negative (responders *n* = 14, non-responders *n* = 9). *p* < 0.05 considered significant.

The variation in levels of each target miRNA by response to NACT was assessed in the indicated breast cancer subtypes. Considering only the luminal subtype, a significant decrease in the miR-21 level is observed in responders compared to non-responders (*p* = 0.048) (Figure 4A). No significant difference in miR-21 levels was observed based on the response to NACT within any of the other three subtypes. For miR-145, there are significantly lower levels in responders compared to non-responders, in luminal breast cancers (*p* = 0.033) (Figure 4B). No significant difference in miR-145 levels was observed based on the response to NACT in any of the other three subtypes. No significant in difference in the levels of Let 7a, miR-195 or miR-155 in response to NACT was observed in any subtype (Figure S3).

## 4. Discussion

In this multicentre, prospective translational trial, we evaluated miRNA levels in serially collected whole blood, for their ability to predict response to standard of care NACT. In this analysis, the circulating levels of five target miRNAs were assessed at the time of diagnosis, prior to NACT, and evaluated in relation to tumour characteristics, biologic features and NACT response. We demonstrated that levels of miR-21 and miR-195, in whole blood at presentation, is altered between NACT responders and non-responders. Using a univariate logistic regression analysis, miRNA-21 was found to be an independent predictor of having a response to NACT. Notably, miR-21 and miR-145 levels had significant variations in levels between responders compared to non-responders in the Luminal breast cancer subtype. These results support recent findings from the NeoALTTO study, which investigated the differentially expressed miRNA isolated from the plasma of Her2+ breast cancer patients. Their study, using two independent cohorts, found a prognostic value of circulating miR-145 (Trastuzumab at week 2; AUC 0.81) and miR-195 (Lapatinib + Trastuzumab at week 2; not included in the final multivariate model) in predicting pCR in Her2+ breast cancer patients [26]. In our study, no significant association was seen between any of the five target miRNAs and any of the breast cancer subtypes, although miR-195 was significantly elevated in OR positive breast cancers, compared to OR negative breast cancers.

MiRNAs have been shown as potential diagnostic biomarkers in breast cancer, with all selected targets in this study shown to be altered in breast cancer patients compared to controls. For both miR-21 and miR-195, it has been established that higher expression levels are associated with breast cancer and poor outcome [17,18,28,29]. Our study not only adds to the growing evidence of the use of miRNA as biomarkers, but is the first to demonstrate that both miR-21 and miR-195 could be potential predictors of response to NACT. Previous studies have also examined the role of miRNA in predicting the response to NACT, and have found that miRNA could be potential biomarkers to predict response. In one study, a group of circulating miRNA were found to exhibit strong correlation with response to NACT [30]. Another study assessing the miRNA levels before and after NACT discovered that a significant variation in miR-34a was seen between patients with a partial response, compared to patients with a complete response in HER2+ (non-luminal) and triple negative breast cancer subtypes [31]. Overall, this highlights the potential of miRNAs alone, or with additional markers, to predict which patients will respond to NACT.

In our study, a breast cancer subtype specific response to NACT was also identified, with elevated miR-21 and miR-145 levels significantly associated with non-responders compared to responders in the luminal breast cancer subtype. For in vitro studies, an elevated miR-21 expression has been shown to be associated with chemoresistance in a luminal breast cancer cell line [32]. In another study, the ability of circulating miRNA to predict response to NACT in luminal breast cancers was assessed, and it was found that circulating miR-19a and miR-205 in serum may predict for chemosensitivity versus chemoresistance in the luminal breast cancer subtype [33]. The luminal breast cancer subtype is known to have the lowest levels of response to NACT; although there are small numbers in our study, we have shown the possibility of using miRNA for identifying which luminal breast cancers will respond to NACT, and further validation with a larger cohort of patients is warranted.

The miRNA targets investigated were selected for this study based on the current published data during the study design. The use of predefined miRNA may have limited the potential of this study, with multiple new targets identified, since it could not be assessed in this study. A recent study found up to 48 publications on circulating miRNA in breast cancer alone [34]. Our work advances this by confirming that specific whole blood isolated miRNA are viable markers for pCR. The lowest rates of recurrence have been seen in luminal cancers that have a pCR [35]. By combining miRNA with clinical and pathological markers, this may increase specificity, and using a combination of markers, it may be possible to make a predictive score for response to treatment, resulting in a more personalized approach to patient care.

## 5. Conclusions

This study has shown again that miRNA are readily detectable in the circulation of breast cancer patients, and for the first time, their potential as biomarkers in predicting response to standard of care NACT. Using a multicentre, prospective translational trial, the ability of a panel of five pre-selected target miRNAs to predict response to standard of care NACT was assessed. Importantly, miR-21 and miR-195 were shown to have significantly reduced levels in patients that responded to standard of care NACT. Furthermore, an intrinsic breast cancer subtype specific to the significant variation in miR-21 and miR-145 expression levels was found to be a predictor of response to NACT. Using a combination of miRNAs alone, or in addition with other clinicopathological factors, may provide an accurate test to assess which patients will benefit from NACT. In this analysis, the miRNA levels at time of diagnosis was used to assess response to standard of care NACT, and may provide a way, in the future, to select the appropriate therapy for patients.

## Figures and Tables

**Table 1:** Target and control miRNA panel and published association with breast cancer.

miRNA of Interest	Previous Association with Breast Cancer
Let 7a	Elevated levels in circulation in breast cancer
miR-21	Increased levels in breast tumour tissue Moreover, increased in: colorectal, pancreatic, gastric, lymphomas
miR-145	Decreased levels in breast tumour tissue
miR-155	Increased levels in breast tumour tissue
miR-195	Increased levels in circulation in breast cancer
miR-16	Validated endogenous circulating control (in breast cancer patients)
miR-425	Validated endogenous circulating control (in breast cancer patients)

**Table 2:** Neoadjuvant chemotherapy patient cohort: Clinicopathological details.

Total Patients Analyzed	*n* = 114	
Median age (range)	55 years (25–76)	
Grade:	*n* = (%)	
1	1	(0.9%)
2	62	(54.4%)
3	50	(43.8%)
Unknown (at time of analysis)	1	(0.9%)
Lymph node (pre op):	*n* = (%)	
Positive	72	(63.2%)
Negative	41	(36.8%)
Surgery:	*n* = (%)	
WLE	63	(55.2%)
Mastectomy	50	(43.9%)
NA	1	(0.9%)
Subtype:	*n* = (%)	
Luminal	57	(49.2%)
Luminal HER2	20	(17.7%)
HER2+	14	(12.9%)
Triple negative	23	(20.2%)
Pathological complete response:	No. (%)	
Yes	51	(44.7%)
No	60	(52.6%)
Unknown	3	(2.6%)

**Table 3:** Relationship of target miRNA on clinicopathological details.

Target miRNA	Grade		Lymph Node Status		OR Status		PR Status		HER2 Status	
	2	3	+Ve	−Ve	+Ve	−Ve	+Ve	−Ve	+Ve	−Ve
Let 7a	*p* = 0.112		*p* = 0.443		*p* = 0.242		*p* = 0.545		*p* = 0.407	
	(*n* = 59, *n* = 50)		(*n* = 70, *n* = 40)		(*n* = 71, *n* = 39)		(*n* = 59, *n* = 52)		(*n* = 32, *n* = 79)	
miR-21	*p* = 0.124		*p* = 0.752		*p* = 0.090		*p* = 0.164		*p* = 0.783	
	(*n* = 61, *n* = 49)		(*n* = 71, *n* = 40)		(*n* = 73, *n* = 39)		(*n* = 59, *n* = 53)		(*n* = 34, *n* = 78)	
miR-145	*p* = 0.968		*p* = 0.075		*p* = 0.406		*p* = 0.063		*p* = 0.877	
	(*n* = 60, *n* = 48)		(*n* = 71, *n* = 38)		(*n* = 71, *n* = 39)		(*n* = 58, *n* = 52)		(*n* = 32, *n* = 78)	
miR-155	*p* = 0.217		*p* = 0.621		*p* = 0.483		*p* = 0.986		*p* = 0.593	
	(*n* = 60, *n* = 50)		(*n* = 71, *n* = 40)		(*n* = 73, *n* = 39)		(*n* = 59, *n* = 53)		(*n* = 34, *n* = 78)	
miR-195	*p* = 0.016		*p* = 0.252		*p* = 0.014		*p* = 0.580		*p* = 0.477	
	(*n* = 61, *n* = 49)		(*n* = 71, *n* = 40)		(*n* = 74, *n* = 38)		(*n* = 59, *n* = 53)		(*n* = 32, *n* = 80)	

## Supplementary Materials

The following are available online at https://www.mdpi.com/2072-6694/12/7/1820/s1, Figure S1: Study Enrollment and analysis Flow Diagram, Figure S2: Univariate analysis of Let 7a, miRNA-145 and miRNA-155 expression as an independent predictor of response, Figure S3: Variation in expression of each target miRNA by response to NACT was assessed in the four breast cancer subtypes, Table S1: Diagnostic accuracy of miRNA and complete response.
