# Supplementary material for: Prospective Assessment of Systemic MicroRNAs as Markers of Response to Neoadjuvant Chemotherapy in Breast Cancer
Source: Cancers (Basel). 2020 Jul 7;12(7):1820. doi: 10.3390/cancers12071820 (PMC7408914; doi:10.3390/cancers12071820)
Supplement: Supplementary file 1 [file cancers-12-01820-s001.pdf]

Supplementary Material: Prospective Assessment of Systemic MicroRNAs as Markers of Response to Neoadjuvant Chemotherapy in Breast Cancer

Andrew McGuire, Maire-Caitlin Casey, Ronan M. Waldron, Helen Heneghan, Olga Kalinina, Emma Holian, Ailbhe McDermott, Aoife J. Lowery, John Newell, Róisín M. Dwyer, Nicola Miller, Maccon Keane, James A.L. Brown and Michael J. Kerin

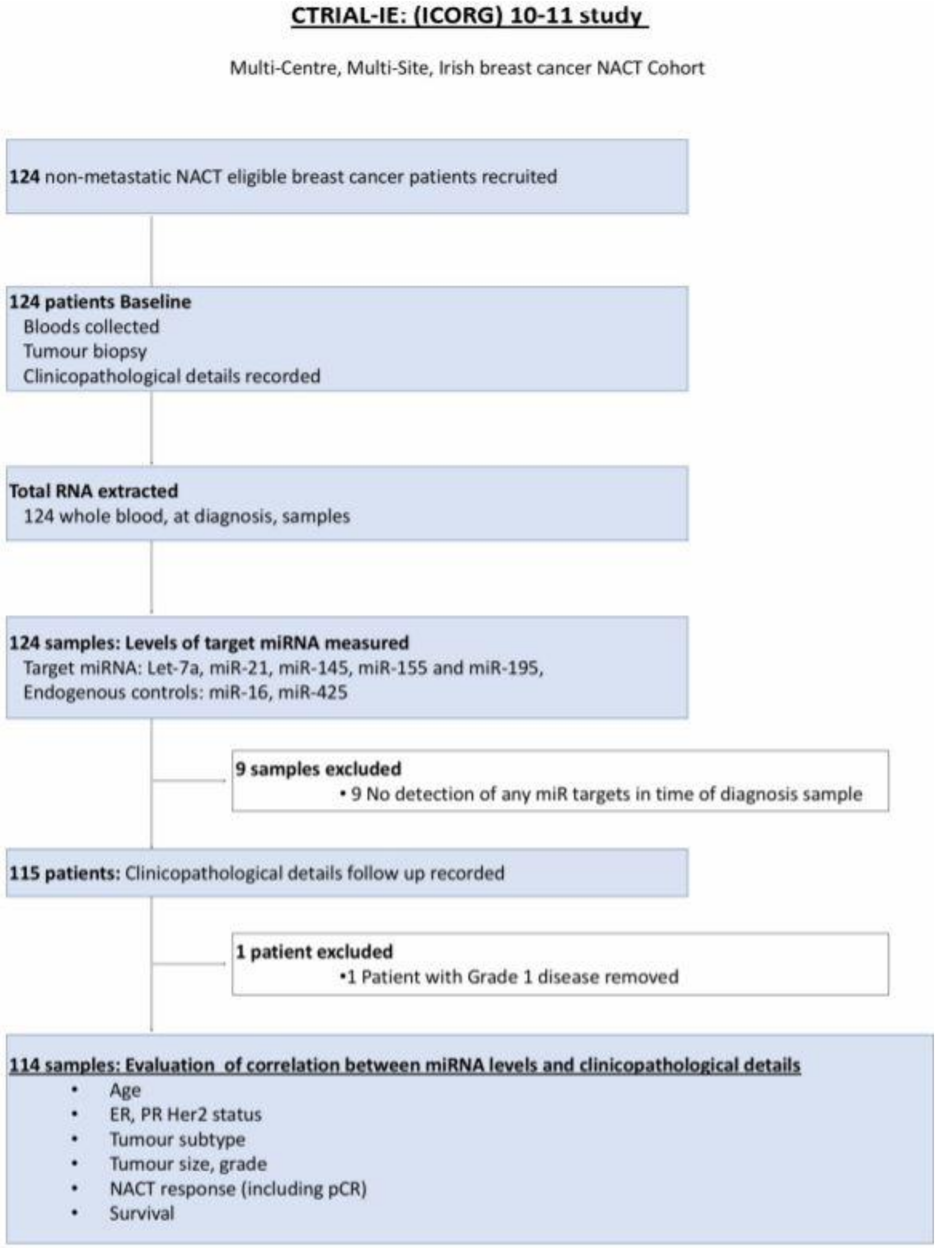

Figure S1. Study Enrollment and analysis Flow Diagram. Illustrates cohort recruitment and sample analysis workflow. Final n = 114 patient samples analyzed.

A

Odds Ratio (95 CI)

	Dependent variable:
	Responders
Let.7a	0.874 (0.522, 1.463)
Constant	0.820 (0.559, 1.202)
Observations	108
R^2	0.003
χ^2	0.264 (df = 1)
Note:	*p<0.1; **p<0.05; ***p<0.01

B

Odds Ratio (95 CI)

	Dependent variable:
	Responders
miR.145	1.004 (0.522, 1.931)
Constant	0.845 (0.578, 1.236)
Observations	107
R^2	0.00000
χ^2	0.0001 (df = 1)
Note:	*p<0.1; **p<0.05; ***p<0.01

C

Odds Ratio (95 CI)

	Dependent variable:
	Responders
miR.155	0.954 (0.603, 1.507)
Constant	0.873 (0.596, 1.280)
Observations	109
R^2	0.001
χ^2	0.042 (df = 1)
Note:	*p<0.1; **p<0.05; ***p<0.01

Figure S2. Univariate analysis of Let 7a, miRNA-145 and miRNA-155 expression as an independent predictor of response. (**A**) Let 7a. (**B**). miRNA-145. (**C**) miRNA-155. Observations = *n* (indicated for each).

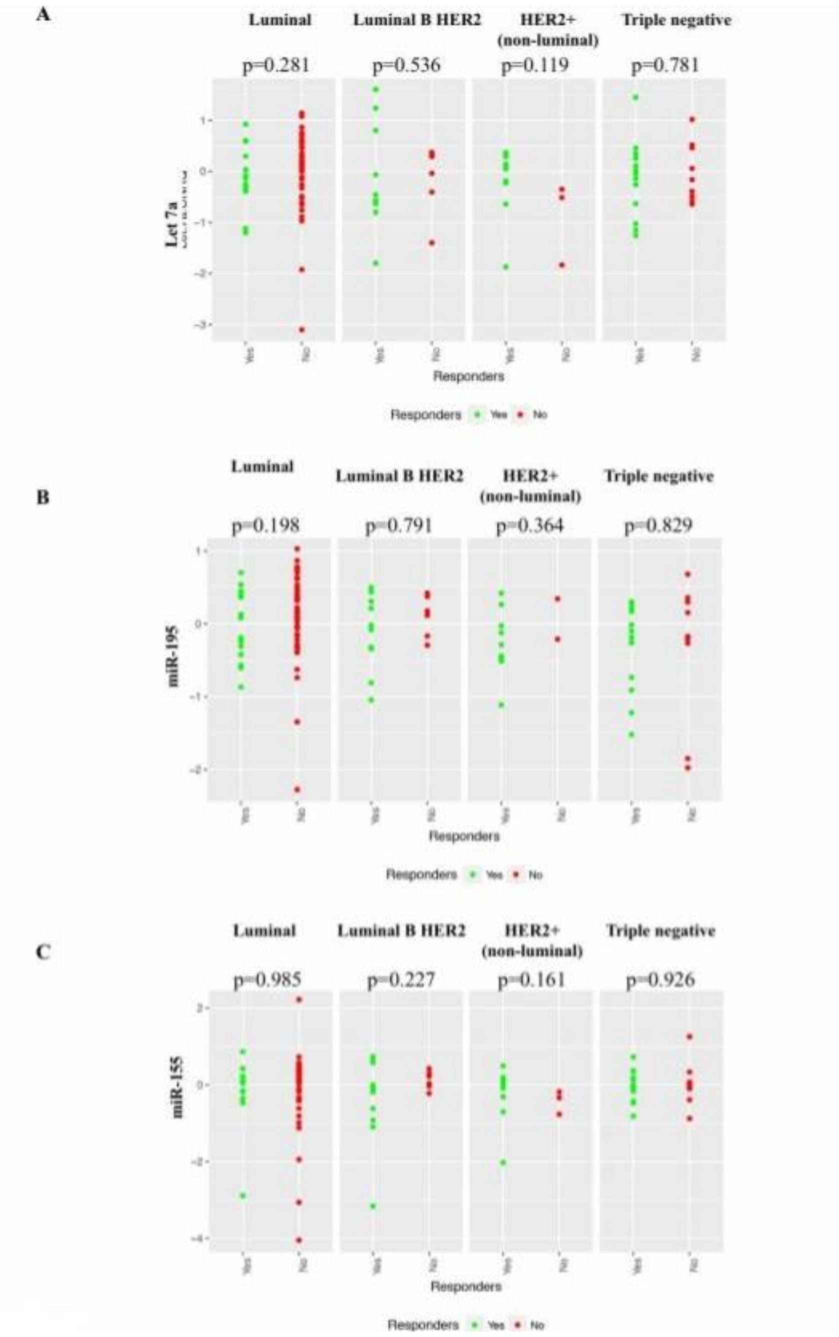

Figure S3. Variation in expression of each target miRNA by response to NACT was assessed in the four breast cancer subtypes. (**A**) Let 7a. (**B**) miR-195. (**C**) miR-155. Luminal (responders n = 15, non-responders n = 41), Luminal B Her2 (responders n = 12, non-responders n = 7), Her2+ (non-luminal) (responders n = 10, non-responders n = 3), Triple negative (responders n = 14, non-responders n = 9). $p < 0.05$ considered significant.

Table S1. Diagnostic accuracy of miRNA and complete response.

Target miRNA	Complete Response AUC (Sensitivity, Specificity)
Let-7a	56.8% (45.2%, 68.5%)
miR-21	58.3% (46.7%. 69.9%)
miR-145	51.5% (39.4%, 63.6%)
miR-155	53.9% (42.5%, 65.3%)
miR-195	67.1% (56.0%, 78.3%)
